# Successful treatment of Depot Medroxyprogesterone acetate-related vaginal bleeding improves continuation rates in Adolescents

**DOI:** 10.1100/tsw.2006.69

**Published:** 2006-03-19

**Authors:** Kristin M. Rager, Amy Fowler, Hatim A. Omar

**Affiliations:** Department of Pediatrics, Section of Adolescent Medicine, University of Kentucky Medical Center, Lexington, KY 40536, USA

**Keywords:** vaginal bleeding, adolescents, depot medroxyprogesterone, DMPA, United States

## Abstract

High discontinuation rates for depot medroxyprogesterone acetate (DMPA) in adolescents may contribute to the number of unintended pregnancies. Many cite vaginal bleeding as a reason for discontinuing DMPA use. In this study, we attempted to determine if treating DMPA-associated vaginal bleeding with monophasic oral contraceptive pills (OCP) raised continuation rates. A total of 131 patients who reported vaginal bleeding while on DMPA were included in this study and 83 were treated with monophasic OCP. Of those who received OCP, 38.7% reported that vaginal bleeding stopped completely, 51.8% reported that vaginal bleeding stopped temporarily, and 6.0% reported no change. Overall, 94% of enrolled patients who received OCP as a treatment for DMPA-associated vaginal bleeding continued DMPA use. Our findings indicate that vaginal bleeding due to DMPA can be successfully treated, leading to improvement in continuation rates.

## INTRODUCTION

In a recent prospective study in adult women, continuation rates for depot medroxyprogesterone acetate (DMPA) were reported to be 68, 67, and 55% at 6, 9, and 12 months. respectively[1]. In adolescents, DMPA continuation rates were even lower. O’Dell et al.[2] found a continuation rate of 34% in postpartum adolescents at 12 months. Zibners et al.[3] reported a 45% continuation rate after 1 year in adolescent DMPA users attending a midwest urban health clinic[3], and Potter et al.[4] found a 35% continuation after 1 year among adolescents attending a rural contraceptive clinic in North Carolina.

Menstrual changes and prolonged bleeding are common reasons for discontinuation of DMPA[5]. Menstrual irregularity was reported by 22% of physicians as the reason patients cited for discontinuation of DMPA[6]. Ammerman[7] found that 33% of patients experienced irregular menses or breakthrough bleeding on DMPA, which is a rate similar to our finding of 32% in 2002[8].

We performed a retrospective study to evaluate whether treatment with combined oral contraceptive pills (OCP) decreased reported bleeding in those on DMPA. A further question was to assess the effect of decreased bleeding on continuation rates. As DMPA is highly efficacious (with 0.3 pregnancies per 100 woman-years vs. 5 pregnancies per 100 woman-years with OCP, and 20 pregnancies per 100 woman-years with male condoms) and easy to use, increasing rates of continuation in adolescents may decrease the number of unintended pregnancies[9].

## METHODS

A retrospective review of data was performed utilizing patient charts from a university-based adolescent medicine clinic. Patients who were started on DMPA on or after January 1996 were included (n = 538). Mean age was 20 years (range 14–20 years) and 56.5% were African-American (see Table 1). Patients who reported significant vaginal bleeding (requiring pad/tampon change more than three times a day for greater than 1 week) while on DMPA were started on a monophasic OCP containing 250 mcg norgestimate and 35 mcg ethinyl estradiol (Ortho-cyclen, Ortho-McNeil Pharmaceuticals, Inc). Patients were given one pill by mouth twice a day for 10 days. Those who reported continued significant bleeding following 10 days of treatment were treated for 10 additional days. Patients were followed for 18 months to assess continuation rates.

**TABLE 1**
**Demographic Characteristics**

| Age | | Race | | Insurance | |
|---|---|---|---|---|---|
| Mean age | 20 years | White | 43.5% | Medicaid | 62.6% |
| Standard deviation | 1.85 years | African-American | 56.5% | None | 13.7% |
| Median | 20 years | | | Private | 6.9% |
| Age range | 14–22 years | | | Unknown | 16.8% |

## RESULTS

Vaginal bleeding was reported by a total of 131 (24.3%) women on DMPA in our study; 41% of the patients reported bleeding after only one injection, while 29.8% reported continued bleeding after the second injection. Of patients reporting significant vaginal bleeding, 83 (63.4%) received OCP. In those patients given OCP, 38.6% reported that bleeding was completely halted. Vaginal bleeding was temporarily suspended in 51.8%, and 6.0% reported that bleeding did not cease at all. A single course of OCP was successful in stopping or significantly reducing vaginal bleeding in 47%, while 26.5% required two courses of treatment.

Continuation rate for DMPA in our clinic overall was 71% at 18 months. Among those with vaginal bleeding who received treatment with OCP, we found a 94% continuation rate at 18 months. Of note, the 6% who were not still on DMPA at 18 months were the same 6% who reported no improvement in vaginal bleeding with the OCP treatment.

## DISCUSSION

Discontinuation of contraceptive method may lead to unintended pregnancies in adolescents. DMPA has historically had poor continuation rates[1,2,3,4], which is unfortunate in light of its superior efficacy[9]. We found that our clinic sample has a higher-than-average continuation rate at baseline (71%). We believe this to be secondary to the comprehensive health care that is provided in our clinic[8]. This number is still unacceptable, as discontinuation leads to lack of protection from pregnancy. As vaginal bleeding is a common reason for discontinuation, we may infer that in decreasing this common side effect, we were successful in achieving significant improvement in continuation rates for DMPA at 18 months.

## CONCLUSION

Decreasing DMPA-associated vaginal bleeding through the use of combination OCP can improve continuation rates among adolescents. Improved contraceptive continuation may decrease the rate of unintended adolescent pregnancy.

## BIOSKETCHES

**Kristin Rager, MD, MPH,** Assistant Professor of Pediatrics, Section of Adolescent Medicine, Department of Pediatrics, University of Kentucky, Lexington. E-mail: rager@uky.edu.

**Amy Fowler,** Graduate Student, School of Public Health, University of Kentucky, Lexington. E-mail: alfowl2@uky.edu

**Hatim A. Omar, MD,** Professor of Pediatrics and Obstetrics and Gynecology and Director of the Section of Adolescent Medicine, Department of Pediatrics, University of Kentucky, Lexington. Dr. Omar has completed residency training in obstetrics and gynecology as well as pediatrics. He has also completed fellowships in vascular physiology and adolescent medicine. He is the recipient of the Commonwealth of Kentucky Governor's Award for Community Service and Volunteerism and is well known internationally with numerous publications in child health, pediatrics, adolescent medicine, pediatric and adolescent gynecology. E-mail: haomar2@uky.edu.
